# Spatial diversity of spontaneous activity in the cortex

**DOI:** 10.3389/fncir.2015.00048

**Published:** 2015-09-24

**Authors:** Andrew Y. Y. Tan

**Affiliations:** Center for Perceptual Systems and Department of Neuroscience, The University of Texas at AustinAustin, TX, USA

**Keywords:** spontaneous activity, cortex, fixation, slow oscillations, attention, plasticity, reinforcement learning

## Abstract

The neocortex is a layered sheet across which a basic organization is thought to widely apply. The variety of spontaneous activity patterns is similar throughout the cortex, consistent with the notion of a basic cortical organization. However, the basic organization is only an outline which needs adjustments and additions to account for the structural and functional diversity across cortical layers and areas. Such diversity suggests that spontaneous activity is spatially diverse in any particular behavioral state. Accordingly, this review summarizes the laminar and areal diversity in cortical activity during fixation and slow oscillations, and the effects of attention, anesthesia and plasticity on the cortical distribution of spontaneous activity. Among questions that remain open, characterizing the spatial diversity in spontaneous membrane potential may help elucidate how differences in circuitry among cortical regions supports their varied functions. More work is also needed to understand whether cortical spontaneous activity not only reflects cortical circuitry, but also contributes to determining the outcome of plasticity, so that it is itself a factor shaping the functional diversity of the cortex.

## Introduction

The neocortex is a layered sheet across which a basic organization is thought to widely apply (Douglas et al., 2003). Excitatory and inhibitory connectivity within each layer is local (Perin et al., 2011; Levy and Reyes, 2012), and excitatory information flows into layer 4 of the cortex, to the superficial layers, then the deep layers (Thomson and Lamy, 2007). The local connectivity within a layer and vertical information flow across layers enables the heuristic of a columnar unit of computation repeated across the cortical sheet, historically termed a “minicolumn”.

However, the basic organization is only an outline which needs adjustments and additions. For example, layer 6 also receives direct thalamic input, such that its latencies in the cat primary auditory cortex can be shorter than those of the superficial layers (Atencio et al., 2009), and even comparable in rodent primary auditory and somatosensory cortices to those of layer 4 (Sugimoto et al., 1997; Constantinople and Bruno, 2013). The modifications to the basic organization must vary spatially, because cortical areas differ in cytoarchitecture, and receive different inputs (Markov et al., 2014; Oh et al., 2014). Some areas may deviate substantially from the basic organization. For example, the presence of layer 4 in motor cortex is debated (Kaneko, 2013). The idea of a basic organization is thus widely acknowledged, but its most fruitful definition and range of applicability remain open (Harris and Shepherd, 2015). Computational models support the notion that varied repetition of a basic organization can explain a wide range of cortical functions (Buonomano and Merzenich, 1995; Ardid et al., 2007; Serre et al., 2007; Bengio et al., 2015).

**Figure 1:**
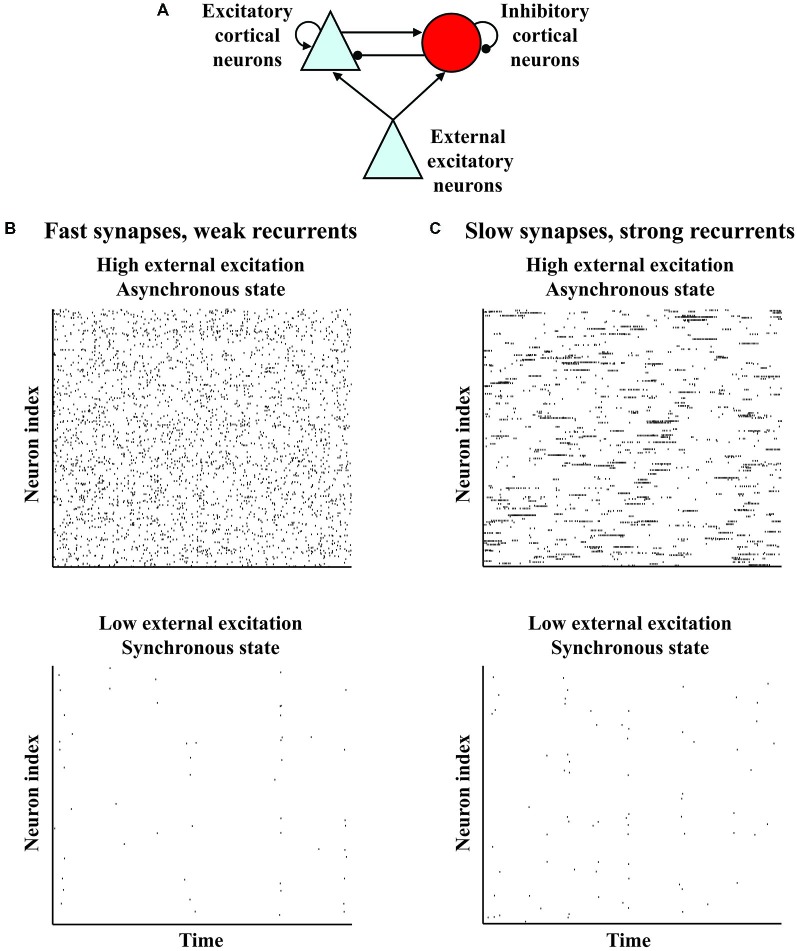
**Variant models of a basic organization show variants of a common behavior in which external excitation shifts the network from a synchronous to an asynchronous state. (A)** Basic organization of models of a small patch of a cortical layer. The model networks contain recurrently connected excitatory and inhibitory neurons which receive external excitation. **(B)** Rasters indicating spike times of neurons from a model network (van Vreeswijk and Sompolinsky, 1998; Brunel, 2000; Mehring et al., 2003; Renart et al., 2010). The neurons show some sychronization at low external excitation, but become asynchronous at high external excitation. **(C)** Rasters indicating spike times of neurons from a variant model network with stronger recurrent connections and more slowly decaying synapses (Harish and Hansel, 2015). Like the model network in **(B)**, the neurons of the variant show some synchronization at low external excitation, but become asynchronous at high excitation. However, this variant model network differs from the model network in **(B)**, because each neuron of this variant in the asynchronous state has periods of sustained firing such that the autocorrelation of the neuron decays more slowly.

Spontaneous activity is neural activity that is present even when all of a set of conventional variables are held constant, as is typically done in a reference or baseline state. It indicates initial variability, which together with the dynamics determines response variability (Kisley and Gerstein, 1999; Curto et al., 2009), and may influence plasticity (Legenstein et al., 2008; Toyoizumi et al., 2013; Chaisanguanthum et al., 2014). Spontaneous activity depends on behavioral state, and is present except in the most pathological conditions (Buzsáki, 2006; Wang, 2010; Ganzetti and Mantini, 2013). Across wide swaths of cortex, activity observed electroencephalographically (EEG) or via local field potential (LFP) in a quieter behavioral state (which may serve as a baseline state) often exhibits lower frequency power, which diminishes in a more active behavioral state; there may also be increased higher frequency power in the more active state (Buzsáki, 2006; Harris and Thiele, 2011). For example, the preponderance of slow oscillations (0.5–4 Hz) in deep non-rapid-eye-movement sleep decreases and is accompanied by increased alpha power (8–12 Hz) when a person awakens (Brown et al., 2010). Analogously, alpha power often decreases upon sensory stimulation or movement initiation (Buzsáki, 2006), while gamma power (30–80 Hz) often increases with alertness, visual stimulation or attention (Gray et al., 1989; Fries et al., 2001; Buzsáki, 2006; Harris and Thiele, 2011). Because the EEG and LFP represent many neurons, the decrease in lower frequency power suggests that sensory stimulation or attention desynchronizes the lower frequency “noise” correlations of nearby neurons, as has been widely observed (de Oliveira et al., 1997; Fries et al., 2001; Kohn and Smith, 2005; Cohen and Maunsell, 2009; Mitchell et al., 2009; Oram, 2011; Smith and Sommer, 2013; Tan et al., 2014). Crucially, models of a small patch of a cortical layer, based on data from cortical slices (Figure 1A), have a robust regime in which external excitation shifts the network from synchrony to asynchrony (Figure 1B) and increases the frequency at which synchrony peaks (van Vreeswijk and Sompolinsky, 1998; Brunel, 2000; Mehring et al., 2003; Renart et al., 2010; Tan et al., 2014). Thus, key aspects of a common pattern in the variety of spontaneous activity occurring with shifts in behavioral state are captured by a basic organization.

However, because the basic organization needs modification according to cortical area, spontaneous activity is presumably spatially diverse, even within a behavioral state. For example, primate prefrontal cortex (PFC) neurons have more spines than primary visual cortex (V1) neurons (Elston, 2000). By assuming that spine number indicates recurrent connectivity strength, Chaudhuri and colleagues argue that PFC and V1 spontaneous activity differ (Chaudhuri et al., 2015). This possibility can be understood by noting that a variant of the basic organization with stronger recurrent connections and more slowly decaying synapses suggested by prefrontal N-methyl D-aspartate receptor 2B subunits (Wang et al., 2013) exhibits the shift from synchrony to asynchrony, but with autocorrelations in the asynchronous state that decay more slowly (Figure 1C; Chaudhuri et al., 2015; Harish and Hansel, 2015).

Accordingly, this review surveys laminar and areal diversity in cortical spontaneous activity that might be expected because there is structural and functional diversity across the cortex. Behavioral state affects spontaneous activity, so we use it to structure our comparisons. We shall be interested in the cortical distribution of spontaneous activity within particular behavioral states, as well as how the distribution is affected by shifts in behavioral state. We begin with broadly-defined behavioral states that are nonetheless sufficiently specified for some spatial comparisons: fixation, an alert state in which gaze is held steady; as well as quieter states of sleep and anesthesia in which slow oscillations are present. Fixation defines a behavioral state broadly, because it can be performed in various attentional contexts. Hence, we will go on to consider more refined behavioral state specifications by examining the effects of attention, anesthesia and plasticity on the cortical distribution of spontaneous activity.

## Laminar and Areal Diversity of Spontaneous Activity During Fixation

Spontaneous activity in macaque V1 varies by layer during fixation. It alternates between low and high, being low in layers 2/3, 4B and 5, and high in layers 4A, 4C and 6 (Poggio et al., 1977; Snodderly and Gur, 1995; Gur et al., 2005). Additionally, layer 2 has more spontaneous activity than layer 3 (Gur and Snodderly, 2008). Spontaneous activity is affected by ambient light level (Kayama et al., 1979), but its laminar pattern is similar in dark and light (Snodderly and Gur, 1995). Stimulus-evoked cross-correlations vary with V1 layer, but spontaneous cross-correlations do not (Hansen et al., 2012).

The expectation that spontaneous activity is spatially diverse is based in part on functional differences among cortical areas, which may therefore indicate features useful for characterizing the spatial diversity of spontaneous activity. A feature that functionally distinguishes cortical areas is the time scale over which a stimulus affects subsequent activity. Sensory cortical responses prominently decay nearly to baseline within several hundred milliseconds after the end of a stimulus, although appropriate tests show that longer time scales are also present (Fishman et al., 2001; Super et al., 2001; Micheyl et al., 2005). In contrast, the firing rates of prefrontal neurons can remain elevated for several seconds after the end of a stimulus, during the delay period of a working memory task (D’Esposito and Postle, 2015), with many features of the delay period activity compactly captured by attractor networks (Wimmer et al., 2014).

Are the different time scales that functionally distinguish cortical areas reflected in spontaneous activity? The autocorrelation of spontaneous activity decays more slowly in the frontal eye field (FEF) than in visual area V4 (Ogawa and Komatsu, 2010). Across neurons in the lateral parietal area (LIP), the time scale of autocorrelation decay correlates positively with the selectivity of delay period activity for target location (Nishida et al., 2014). Furthermore, the autocorrelation of fluctuations of spontaneous activity from the trial average decays more slowly from the medial temporal area (MT) to LIP to the lateral PFC (LPFC) and orbitofrontal cortex (OFC) to the anterior cingulate cortex (ACC; Murray et al., 2014). Across LIP, LPFC and ACC, the time scale over which the autocorrelation of fluctuations decays correlates positively with the time scale over which a reward in one trial influences neural activity in subsequent trials (Murray et al., 2014). The variety of time scales may be due to each area’s position in the cortical hierarchy and factors intrinsic to each area (Murray et al., 2014; Chaudhuri et al., 2015).

It is important to keep in mind that the context in which fixation is performed affects spontaneous activity (Andersen et al., 1990; Colby et al., 1996). However, the details of the above-mentioned studies indicate that the laminar differences in V1 and the slower decay of autocorrelations at higher cortical levels are robust across fixation in several contexts.

## Laminar and Areal Diversity of Slow Oscillations

Cortical slow oscillations are present during deep non-rapid-eye-movement sleep (Steriade et al., 2001; Buzsáki, 2006), ketamine and urethane anesthesia (Fuster et al., 1965; Fox and Armstrong-James, 1986; Metherate and Ashe, 1993; Steriade et al., 1993), and in cortical slices (Sanchez-Vives and McCormick, 2000). During slow oscillations, the EEG exhibits large amplitude fluctuations and the membrane potentials of cortical neurons alternate between depolarized “up” states and hyperpolarized “down” states (Fuster et al., 1965; Metherate and Ashe, 1993; Steriade et al., 1993). Increased activity during a down-to-up transition first occurs in layer 5 in cortical slices (Sanchez-Vives and McCormick, 2000), in the deep layers of the auditory cortex of urethane-anesthetized rats (Sakata and Harris, 2009) and the suprasylvian areas of ketamine-anesthetized and sleeping cats (Chauvette et al., 2010). In the sensory-motor areas of urethane-anesthetized mice slow oscillations are more greatly attenuated by suppressing the deep than the superficial layers (Beltramo et al., 2013). The prominence of the deep layers in slow oscillations in slices and *in vivo* suggests they are generated by common mechanisms, but the extent to which this is the case remains unclear (Crunelli et al., 2014). In contrast to the aforementioned studies, current source density analyses of recordings during sleep from frontal and parietal cortical areas of patients with drug-resistant focal epilepsy suggest that the superficial cortical layers are important for generating slow oscillations (Csercsa et al., 2010).

There is considerable diversity in the synchrony between slow oscillations in different cortical areas within the same hemisphere of urethane-anesthetized mice, although bilaterally corresponding regions are more synchronous (Mohajerani et al., 2010). Such diversity is also present during and varies over the course of human sleep, with fewer cortical regions exhibiting slow waves later into sleep (Nir et al., 2011). Asynchrony may be due to a consistent non-zero phase difference, or a lack of any consistent phase relationship. Both possibilities occur. In ketamine-anesthetized guinea pigs, slow oscillations in corresponding frequency regions of different tonotopic areas are coherent, having consistent phase differences that parallel the sound-evoked latencies of the various areas, whereas slow oscillations of different frequency regions are incoherent (Farley and Noreña, 2013). Slow oscillations thus resemble spontaneous gamma activity in reflecting auditory tonotopic organization (Fukushima et al., 2012).

Spontaneous activity can also distinguish between tonotopic and non-tonotopic areas, as the tonotopic primary auditory and non-tonotopic dorsoposterior (DP) fields of ketamine-anesthetized mice are distinguished by prominent spontaneous pulses in DP that can be entrained by sound (Stiebler et al., 1997; Joachimsthaler et al., 2014). Slow oscillations in ketamine-anesthetized mice further distinguish the PFC from the primary visual, somatosensory, and motor cortices, as PFC slow oscillations have faster down-to-up state transitions, higher firing rates during up states, and more regular cycles (Ruiz-Mejias et al., 2011).

## Modulation of Spontaneous Activity by Attention and Anesthesia

Each previous section focused on spontaneous activity within a particular broadly-defined behavioral state. Since spontaneous activity is modulated by behavioral state, it is also interesting to compare its distribution across behavioral states. Accordingly we turn now to the effects of varying attentional context and anesthetic depth on the distribution of spontaneous activity in the cortex.

An animal may perform a reference behavior such as fixation following various cues, each of which sets an attentional context by signaling information about the task to be performed after the reference behavior. The neural activity during the reference behavior is conventionally called “spontaneous” or “baseline” activity (Luck et al., 1997; Chawla et al., 1999; Recanzone and Wurtz, 2000). Its distribution can be cue-dependent. For example, after monkeys viewed a cue indicating a particular visual location, baseline activity differentially increased in extrastriate visual neurons selective for the cued location (Luck et al., 1997; Recanzone and Wurtz, 2000). Similarly, after human subjects received a cue indicating either motion or color (Chawla et al., 1999), target location or color (Giesbrecht et al., 2006), a particular stimulus modality (Saupe et al., 2009; Langner et al., 2011) or object category (Puri et al., 2009), baseline activity differentially increased in cortical areas selective for the cued feature. The tasks in these studies involved attention as well as working memory, and the cue-dependence of baseline activity is thought to involve top-down signals from frontal and parietal cortical areas (Beck and Kastner, 2009).

Anesthetic depth also modulates the distribution of spontaneous activity. Several anesthetics cause EEG anteriorization in humans, in which EEG power shifts from posterior to anterior electrodes (Brown et al., 2010). Propofol anteriorization occurs mainly in the alpha range (Feshchenko et al., 2004; Purdon et al., 2013). Propofol has been proposed to increase anterior alpha power by potentiating GABAergic inhibition in the frontal thalamocortical network, and to decrease posterior alpha power by inhibiting the hyperpolarization-activated current I_h_ in the posterior thalamic network (Vijayan et al., 2013). Isoflurane, sevoflurane and halothane act like propofol on the model and were accordingly predicted to cause alpha anteriorization (Vijayan et al., 2013). Sevoflurane does cause the predicted alpha anteriorization, but with a theta coherence not observed with propofol (Akeju et al., 2014). The spatial modulation of spontaneous activity by isoflurane anesthesia has been studied in ferrets (Sellers et al., 2013). Spontaneous LFP in V1 of awake ferrets in a dark room exhibited a spectral peak near 18 Hz in the deep layers, which shifted towards 10 Hz with increasing isoflurane concentration. In comparison, spontaneous LFP in the PFC exhibited increased power at all frequencies in all layers, and developed a spectral peak near 10 Hz in layer 4 and the deep layers with increasing isoflurane concentration. At the highest concentration the 10 Hz peak was abolished in V1 but sustained in the PFC, a pattern reminiscent of alpha anteriorization in humans. Anteriorization and the diversity of autocorrelation time scales (discussed in the section on fixation) both indicate differences between occipital and frontal areas, but it remains to be understood whether they are due to the same differences in circuitry.

## Modulation of Spontaneous Activity by Plasticity

Longer lasting circuit modifications are likewise reflected in spontaneous activity. For example, coincident tone presentation and nucleus basalis stimulation, which alters auditory cortical frequency selectivity and enhances perceptual learning (Bakin and Weinberger, 1996; Kilgard and Merzenich, 1998; Reed et al., 2011), increases the spontaneous firing rates of neurons in the primary auditory cortex and posterior auditory field of pentobarbital-anesthetized rats (Puckett et al., 2007). Increased spontaneous firing rates have also been observed in PFC neurons of macaques that had learned a working memory task (Qi et al., 2011). The increased rates were accompanied by decreased baseline variability (Qi and Constantinidis, 2012b), and decreased correlations between fluctuations from the trial-averaged baseline firing of neurons separated by 0.5–1 mm (Qi and Constantinidis, 2012a). The changes occurred mainly in neurons that responded during stimulus-presentation or delay periods of the learned task. On a much larger spatial scale, changes in spontaneous correlations between visual and fronto-parietal areas following visual perceptual learning were revealed by functional magnetic resonance imaging of the blood-oxygen level-dependent (BOLD) signal in humans, and demonstrated to correlate with learning (Lewis et al., 2009). Alterations in the spatial distribution of spontaneous activity in pathologies such as spatial neglect following stroke (He et al., 2007b) and tinnitus following hearing loss (Weisz et al., 2005; Vanneste et al., 2011) have also been identified, and used to help develop candidate treatments (He et al., 2007a; Langguth et al., 2013).

Does the spatial diversity of spontaneous activity merely reflect structural and functional diversity across the cortex, or might it also have functional significance, perhaps contributing to the functional diversity? The latter possibility is hinted at by evidence that spontaneous activity contributes to determining the outcome of plasticity. For example, stimulation delivered in urethane-anesthetized rats to a cortical column increased its spontaneous correlation with a reference column only if the stimulation was in sync with spontaneous activity in the reference column (Erchova and Diamond, 2004), a result consistent with Hebbian plasticity (Fregnac et al., 1988; Jackson et al., 2006). Correlations between spontaneous activity characteristics and the rate or outcome of learning have been demonstrated in mice (Lin et al., 2013), rats (Arduin et al., 2013), monkeys (Sadtler et al., 2014), and humans (Freyer et al., 2013), suggesting some role for spontaneous activity in plasticity. Spontaneous activity can be an obstacle to long-lasting memories (Fusi et al., 2005). Reinforcement learning, on the other hand, is trial-and-error learning requiring variability, and may benefit from spontaneous activity (Mazzoni et al., 1991). Importantly, reinforcement learning can be implemented with biologically plausible models of synaptic plasticity in cortical networks (Bakin and Weinberger, 1996; Kilgard and Merzenich, 1998; Reynolds et al., 2001; Legenstein et al., 2008; Gavornik et al., 2009; Bourjaily and Miller, 2011; Rombouts et al., 2015). It is worth noting that learning may depend nonmonotonically on neural variability, and that neural covariability can be key (Legenstein et al., 2008, 2010). Intriguingly, a model incorporating a learning rule proposed to explain shifts of excitation and inhibition reported in a reinforcement learning paradigm (Froemke et al., 2007; Vogels et al., 2011) suggests that patterned spontaneous activity can stabilize memories (Litwin-Kumar and Doiron, 2014).

## Summary and Future Directions

Let us summarize with an eye towards avenues for exploration. We have seen that cortical spontaneous activity is spatially diverse even within a behavioral state, a reflection of structural and functional diversity across the cortex. We noted theoretical proposals for the synaptic and intrinsic mechanisms underlying the diversity in function and spontaneous activity, which has been surveyed to date largely by observations of extracellular electromagnetic fields or blood-oxygenation. Further tests of the models are needed, and may be provided, for example, by intracellular recordings of membrane potential. During fixation, membrane potential in macaque V1 is far from spike threshold with non-Gaussian fluctuations (Tan et al., 2014). In contrast, some models of frontal areas predict that membrane potential hovers near spike threshold during fixation (Wang, 1999; Lundqvist et al., 2010; Vijayan et al., 2013; Chaudhuri et al., 2015; Harish and Hansel, 2015), resembling that observed in awake, but non-behaving cats (Steriade et al., 2001). Tests of such possible differences in membrane potential between V1 and frontal areas during fixation would help us understand how differences in circuitry among cortical regions supports their varied functions.

We also saw that behavioral state shifts and plasticity cause momentary or longer-lasting circuit changes that are reflected by the spatial distribution of spontaneous activity. Indeed, it is because spontaneous activity is affected by behavioral state and cortical location that this review has been structured according to states, such as fixation by behaving macaques, that are probably sufficiently specified for the diversity due to cortical location to be reproducibly observed. With respect to further characterization of the spatial diversity by intracellular recording of membrane potential, McGinley and colleagues have recently performed such recordings in mice trained to perform tone detection (McGinley et al., 2015). They show that measurements of pupil diameter characterize behavioral state sufficiently to predict when task performance is best, so trained mice may provide another valuable experimental paradigm in which behavioral state can be sufficiently specified for investigating spatial diversity in spontaneous activity. Interestingly, the mice performed best when spontaneous membrane potential in the auditory cortex was relatively hyperpolarized (McGinley et al., 2015), resembling that in V1 during successful fixation by macaques (Tan et al., 2014).

Finally, we noted that spontaneous activity and the associated response variability could contribute to determining the outcome of plasticity. Neuromodulators like acetylcholine therefore perhaps affect plasticity not only by gating it (Gu, 2002; Chubykin et al., 2013; Chun et al., 2013), but also by modulating spontaneous activity and response variability (Zinke et al., 2006; Goard and Dan, 2009; Zhou et al., 2011). There is evidence that variability is needed for or enhances some forms of motor learning in people (Sans-Muntadas et al., 2014; Taylor and Ivry, 2014) and song birds (Woolley and Kao, 2015). Consequently, more work seems warranted to understand whether the variability associated with cortical spontaneous activity has a role in cortical plasticity, so that it is itself a factor shaping the functional diversity of the cortex.

## Conflict of Interest Statement

The author declares that the research was conducted in the absence of any commercial or financial relationships that could be construed as a potential conflict of interest.
